# Fast In-House Next-Generation Sequencing in the Diagnosis of Metastatic Non-small Cell Lung Cancer: A Hospital Budget Impact Analysis

**DOI:** 10.36469/001c.77686

**Published:** 2023-06-26

**Authors:** Ubong Silas, Maximilian Blüher, Antonia Bosworth Smith, Rhodri Saunders

**Affiliations:** 1 Coreva Scientific GmbH & Co. KG, Königswinter, Germany; 2 Coreva Scientific GmbH & Co KG, Königswinter, Germany

**Keywords:** targeted therapy, turnaround time, health economic analysis, hospital costs, genetic testing, return on investment

## Abstract

**Background:** Targeted therapy for cancer is becoming more frequent as the understanding of the molecular pathogenesis increases. Molecular testing must be done to use targeted therapy. Unfortunately, the testing turnaround time can delay the initiation of targeted therapy.

**Objective:** To investigate the impact of a next-generation sequencing (NGS) machine in the hospital that would allow for in-house NGS testing of metastatic non-small cell lung cancer (mNSCLC) in a US setting.

**Methods:** The differences between 2 hospital pathways were established with a cohort-level decision tree that feeds into a Markov model. A pathway that used in-house NGS (75%) and the use of external laboratories (so-called send-out NGS) (25%), was compared with the standard of exclusively send-out NGS. The model was from the perspective of a US hospital over a 5-year time horizon. All cost input data were in or inflated to 2021 USD. Scenario analysis was done on key variables.

**Results:** In a hospital with 500 mNSCLC patients, the implementation of in-house NGS was estimated to increase the testing costs and the revenue of the hospital. The model predicted a 710 060increaseintestingcosts,a1 732 506 increase in revenue, and a $1 022 446 return on investment over 5 years. The payback period was 15 months with in-house NGS. The number of patients on targeted therapy increased by 3.38%, and the average turnaround time decreased by 10 days when in-house NGS was used.

**Discussion:** Reducing testing turnaround time is a benefit of in-house NGS. It could contribute to fewer mNSCLC patients lost to second opinion and an increased number of patients on targeted therapy. The model outcomes predicted that, over a 5-year period, there would be a positive return on investment for a US hospital. The model reflects a proposed scenario. The heterogeneity of hospital inputs and the cost of send-out NGS means context-specific inputs are needed.

**Conclusion:** Using in-house NGS testing could reduce the testing turnaround time and increase the number of patients on targeted therapy. Additional benefits for the hospital are that fewer patients will be lost to second opinion and that in-house NGS could generate additional revenue.

## BACKGROUND

Lung cancer is the leading cause of cancer-related mortality in the United States (US), and non-small cell lung cancer (NSCLC) accounts for about 85% of all the lung cancer cases.^1,2^ In many cases, NSCLC is diagnosed at an advanced stage when it has already metastatized.^3^ Earlier diagnosis is known to improve patient outcomes.^4^ In cases where the patients were not treated, a mortality rate of 13% within 4 weeks has been reported.^4^

In the management of advanced NSCLC, there are 3 major therapy approaches: chemotherapy, immunotherapy, and targeted therapy.^5^ Targeted therapy for NSCLC is becoming more frequent as the understanding of the molecular pathogenesis increases.^6^ Unlike the nontargeted therapies, biomarker testing is a prerequisite for the initiation of targeted therapy. The testing is aimed at identifying the oncogenic drivers that support the sustained growth of cancerous cells.^6^ Although there is a waiting time associated with molecular testing, targeted therapy has been shown to result in better disease management and improved outcomes in metastatic NSCLC.^7^

Next-generation sequencing (NGS) is a type of molecular biomarker testing that provides comprehensive information about the genes known to be associated with oncogenic drivers.^8^ Another conventional form of molecular biomarker testing is single-gene testing, which assesses one gene, unlike NGS, which assesses multiple genes.^9^ The benefits of fast NGS over other molecular biomarker testing are that it delivers all the relevant findings at once, maximizes results from small tissue samples, and incurs a shorter turnaround time.^10–13^

Conventionally, when NGS testing is indicated, the pathologist will send the sample to an external laboratory, after which the attending physicians (oncologist, pathologist) will receive an analysis report. The setup costs for the hospital are minimal. However, the running costs can be high as each analysis will incur a fee,^9^ and the turnaround time for results can vary from 9 to 30 days.^14^ As an alternative, an NGS machine could be introduced within the hospital, subsequently referred to as “in-house NGS.” A turnaround time of 3 days has been reported with fast in-house NGS,^14,15^ and it may provide additional benefits of more customizable analyses.

The investment costs of introducing in-house NGS are substantial.^9^ In the longer term, however, the costs of providing NGS tests may be reduced. This study aims to assess the budget impact of introducing fast in-house NGS testing as an option for the diagnosis of advanced NSCLC from a hospital perspective and answering the question as to whether in-house NGS has the potential to reduce costs and improve patient outcomes.

## METHODS

A budget impact model was designed to compare the economic impact of in-house NGS vs send-out NGS and single-gene testing. The model was constructed from the perspective of a US hospital. It therefore considers the costs that would be accrued by the hospital. The target population were patients with advanced NSCLC for whom NGS testing would be indicated. The intervention was the use of in-house NGS testing, where all testing is done within the hospital. The comparator is the use of external NGS testing centers. The time horizon of the model was 5 years. The study findings are reported according to the CHEERS checklist.^16^

### Model Structure

The model was constructed as a cohort-level decision tree that feeds into a Markov model. This patient cohort is informed by the number of new metastatic NSCLC cases managed at the hospital per year. The model also allows for the entry of new cohorts of patients in the subsequent years. In our base case, for example, in each year of the 5-year time horizon, 500 patients entered the model, with a total of 2500 patients by the end of the model. Therefore, for years 2 to 5, both the surviving patients from the previous years and the new patients were followed.

**Figure 1.**
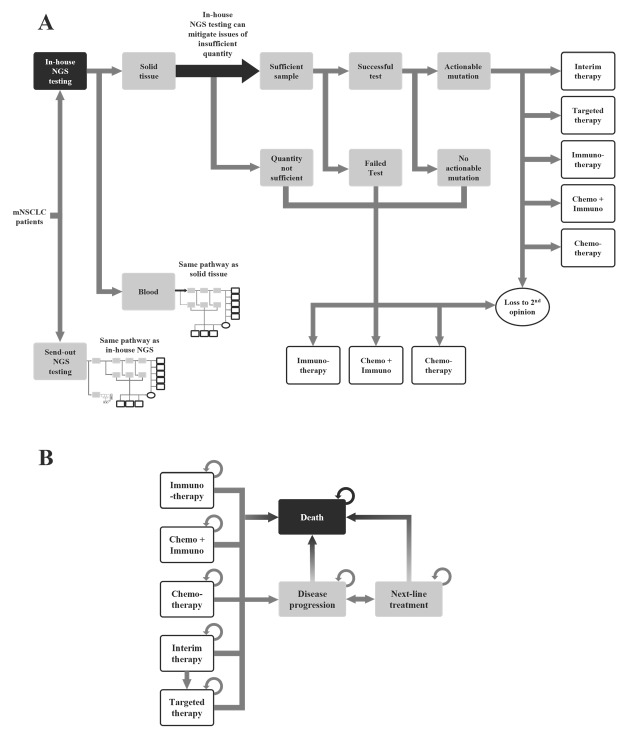
Movement of Patients Through the Decision Tree and Markov Model (**A**) Decision-tree modeling movement of patients through standardized testing pathway until therapy decision; (**B**) Markov model for disease progression from initiation of therapy until death. *Arrows* describe the movement of individuals within and between the different states. Movement to death is possible from any of the states. Abbreviations: chemo + immuno, chemotherapy and immunotherapy; mNSCLC, metastatic non-small cell lung cancer patients.

The model was built in Microsoft Excel. The decision tree represents the movement of patients from testing through to the therapy decision (**Figure 1A**) and accounts for cases when the sample is not sufficient for testing, the test is inconclusive or fails, or the mutation is not actionable. The model accounts for the patients who seek a second opinion, and the model assumes that they will continue treatment at a different hospital. These patients are considered lost to second opinion.

The Markov model represents the progression of the disease from the beginning of the therapy until patient death (**Figure 1B**). The outputs of the decision tree inform the therapy groups in the Markov model. The Markov model allows for the movement of patients between the therapy groups until disease progression or death.

**Standardized testing pathway**: The patients in the hypothetical cohort entered the model once they had been diagnosed and indicated for NGS testing. The patients could have either the standard of care, which is the use of send-out NGS, that is, single-gene testing where the samples are sent to a laboratory outside the hospital for testing, or the use of in-house NGS (**Figure 1A**).

Send-out testing and in-house testing follow a similar pathway. The model considered the type of testing sample, that is, blood or tissue. If there was an insufficient sample or the test was negative or inconclusive, the patients would be started on a nontargeted treatment or the patients could seek out a second opinion at a different cancer center. If the sample was sufficient and the test was successful, the results could either be an actionable mutation or no actionable mutation. When there was no actionable mutation, the patients could start a standard treatment or seek a second opinion. If the mutation was actionable, then the patient could begin with a targeted therapy. The patients could also begin an interim nontargeted therapy until they are switched onto the appropriate therapy after NGS results were received.

The differences between the send-out NGS and in-house NGS were the turnaround time and the availability of sufficient tissue sample. It was assumed that in the in-house NGS scenario, it would be possible to attain more tissue sample. This would allow further testing for in-house NGS when the sample was insufficient. In contrast, the send-out NGS would immediately be in a situation where the testing was unsuccessful, and the patients would move to a nontargeted therapy or to a second opinion.

**Clinical care pathway:** The second part of the model was the Markov model. It contained 8 states: chemotherapy, immunotherapy, chemo + immuno (chemotherapy and immunotherapy), interim therapy, targeted therapy, disease progression, next-line treatment, and death (**Figure 1B**). Once assigned to a therapy in the decision tree, patients could remain on their current therapy or could transition to disease progression. The transition probability of each therapy was informed by the progression-free survival rate of their index drugs. Patients with disease progression would move to next-line treatment and receive a new line of treatment. Patients on interim therapy would move to disease progression or targeted therapy after one cycle.

Patients could die at any time in the model, and the transition probabilities for moving from each state to death was dependent on the transition probabilities and overall survival rate of each therapy. The cycle length was assumed to be 30 days. Testing costs and physician/hospital visits were accounted for as patients moved across the different health states.

**Model input data:** A detailed list of the model inputs and the sources is shown in **Table 1**. The testing data were sourced from a retrospective analysis of real-world data for NSCLC patients.^17^ The therapy decisions and clinical outcome data were sourced from peer-reviewed literature. The cost and revenue-related inputs were based on the external laboratory price list, testing cost, and reimbursement from the Centers for Medicare & Medicaid Services and commercial payers.^18^ All the costs within the model were in or inflated to 2021 USD.

**Table 1.** Model Inputs

**Parameter, Measure**	**Value (Uncertainty)**	**Reference**
NGS testing variables		
Solid tissue sample	60%	Smith et al^17^
Blood sample	40%	Smith et al^17^
Insufficient solid tissue sample	5%	Smith et al^17^
Insufficient blood tissue sample	0.30%	Smith et al^17^
Failed blood test	0.50%	Smith et al^17^
Failed tissue sample test	1.20%	Smith et al^17^
Blood tests with actionable mutation	24%	Smith et al^17^
Tissue sample tests with actionable mutation	23%	Smith et al^17^
Patients on immunotherapy after quantity not sufficient, failed testing and with no actionable mutation	25%^c^	Bains et al^19^
Patients on chemotherapy after quantity not sufficient, failed testing and with no actionable mutation	29%^c^	Bains et al^19^
Patients on chemo + immuno after quantity not sufficient, failed testing and with no actionable mutation	36%^c^	Bains et al^19^
Patients with actionable mutation on targeted therapy	73%^c^	Smith et al^17^
Patients with actionable mutation on interim therapy	27%^c^	Smith et al^17^
Patients switched to target therapy after interim	34%^c^	Smith et al^17^
Cost		
In-house NGS testing, per test	$600 (578, 908)	Sabatini et al^18^
Send-out NGS testing, per test	$300 (270, 330)^a^	List price of laboratories^b^
Single-gene testing, per test	$141	Johnston et al^9^
Acquisition cost of in-house NGS	$200 000	Expert opinion
Revenue		
Reimbursement for in-house NGS testing, per test	$580 (522, 638)^a^	Sabatini et al^18^
Hospital visit	$124 (112, 136)^a^	Vanderpoel et al^13^
Turnaround time, days		
Send-out	10.32-27.80	Sheffield et al,^14^ Smith et al^17^
In-house	3	Sheffield et al,^14^ Ilié et al^15^
Clinical events		
Progression-free survival, 1 year		
Chemotherapy	17.3% (95% CI 12.0-23.5)	Gandhi et al^20^
Immunotherapy	27.9%	Wang et al^21^
Chemo + immuno	34.1% (95% CI 28.8-39.5)	Gandhi et al^20^
Targeted therapy	40% (95% CI 28-52)	Rosell et al^22^
Overall survival, % at 1 year		
Chemotherapy	49.4% (95% CI, 42.1- 56.2)	Gandhi et al^20^
Immunotherapy	67.3%	Wang et al^21^
Chemo + immuno	69.2% (95% [CI] 64.1- 73.8)	Gandhi et al^20^
Targeted therapy	86.0% (95% [CI] 80.7- 89.9)	Mok et al^23^
Disease progression to next-line treatment, % at 3 mo	48%	Davis et al^24^
Next-line treatment to disease progression, % at 3 mo	50%	Davis et al^24^

Model inputs derived from retrospective analysis of real-world data, expert opinions, and peer-reviewed articles.

^a^Assumed variance of 10% was used.

^b^Send-out NGS costs estimated as 10% to 12% of list price of comprehensive genomic profiling testing ($3000).

^c^Proportion from study was weighted using data from Smith et al (2021).^17^

Abbreviations: NGS, next-generation sequencing; chemo + immuno, chemotherapy and immunotherapy.

**Model settings:** There were 2 different hospital scenarios used for the base-case setting of the model: current and proposed. The current scenario was send-out NGS only and the proposed scenario was a mixture of in-house and send-out NGS. The model settings for the hospital base-case parameters can be found in **Table 2**.

**Table 2.** Hospital Base-Case Parameters

**Parameter**	**Value**
Time horizon (years)	5
Annual discount rate (%)	3.50
Population	
Metastatic NSCLC patients per year (n)	500
Total metastatic NSCLC patients for the 5-year time horizon (n)	2500
Current scenario	
Proportion of in-house NGS testing (%)	0
Proportion of send-out NGS testing (%)	100
Proposed scenario	
Proportion of in-house NGS testing (%)	75
Proportion of send-out NGS testing (%)	25

Abbreviations: NSCLC, non-small cell lung cancer; NGS, next-generation sequencing.

### Model Outcomes

The key outcomes of the model included the total testing-related costs and the revenue, the return on investment (ROI), and the payback period of the current and proposed scenarios. The ROI was defined to be the budgetary impact of introducing in-house NGS after a fixed period and payback period as the time to the break-even point.

The other model outcomes were the average NGS result turnaround time, the number of patients retained, and the proportion of patients on targeted therapy for both scenarios.

### Sensitivity and Scenario Analyses

Probabilistic sensitivity analysis was done by running 1000 simulations to test the robustness of the model results. The results of the sensitivity analysis were reported as a 95% credible interval next to the base-case results. An assumed variance of 10% variance was used when the variance of a model input was unavailable; this was informed by the uncertainty range of other model input data extracted from peer-reviewed clinical and economic studies.

A one-way sensitivity analysis was performed to identify the model inputs that have the greatest impact on the model outputs. These results are presented as a tornado diagram.

Given the variability in hospital practice in the US, alternative strategies were explored. This tested the ROI and break-even point with 3 different alterations to the base-case parameters. The first was the increased proportion of in-house NGS testing in the proposed scenario, the second was a change in the number of patients per year, and the third was single-gene testing instead of send-out NGS in the current scenario.

## RESULTS

### Base-Case Results

**Figure 2.**
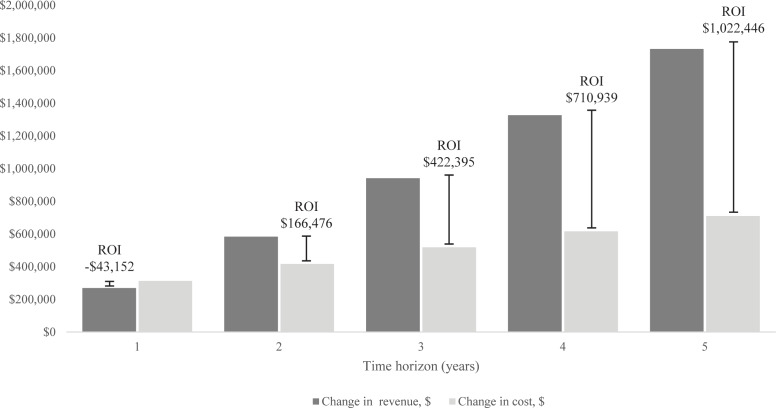
Change in Revenue, Cost, and ROI for Each Year of the Time Horizon Abbreviation: ROI, return on investment.

The results of the current scenario vs the proposed scenario are reported in **Table 3**. The model estimated a total testing cost of $680 080 for the current scenario and $1 390 140 for the proposed scenario. The proposed scenario had an increased cost of $710 060. The proposed scenario also had a greater revenue, and it was estimated that there would be an additional $1 732 506 of revenue over 5 years. The payback period for the proposed scenario was estimated to be 15 months (median: 15.26; 95% confidence interval [CI]: 14, 17) in comparison to the current scenario. The ROI was $1 022 446 (median: $1 008 178; 95% CI: $788 019, $1 250 007) over 5 years at base case with an annual discount rate of 3.5%; the undiscounted ROI was $1 082 136 (median: $1 066 583; 95% CI: $829 991, $1 331 117). The annual results for the ROI can be found in **Figure 2**. The ROI was negative for the first year but positive for the remaining years within the time horizon.

**Table 3.** Model Results at 5-Year Time Horizon

	**Current Scenario**	**Proposed Scenario**	**Difference**
Testing cost ($)	680 080	1 390 140	710 060
Revenue ($)	11 741 432	13 473 938	1 732 506
ROI ($)	NA	1 022 446	–
Payback period (mo)	Vs base case	15	–
Average turnaround time (days)	19	9	-10
Patients on targeted therapy (%)	10.35	13.73	3.38
Patients lost to second opinion (n)	173	64	-109

Abbreviations: NA, not applicable; ROI, return on investment.

The proposed scenario reduced the turnaround time by an estimated 10 days (median: 9.85; 95% CI: 9.13, 10.50 days). The proposed scenario had a turnaround time of 9 days compared with 19 days of the current scenario, a 52.6% relative reduction in turnaround time. In the current scenario, 173 patients were estimated to be lost to second opinion. In the proposed scenario, 109 (63%) of those patients were retained over the 5-year period, with only 64 lost to second opinion. The percentage of patients on targeted therapy increased over the time horizon starting with a 1.86% difference in year 1 to a 3.38% increase in year 5 (see **Online Supplementary Material**). At year 5, the model estimated that the proposed scenario would result in a 3.38%-point (median: +3.3; 95% CI: +2.31, +4.05) increase in the number of patients on targeted therapy at the end of the time horizon, which corresponds to a 32.7% relative increase.

**Figure 3.**
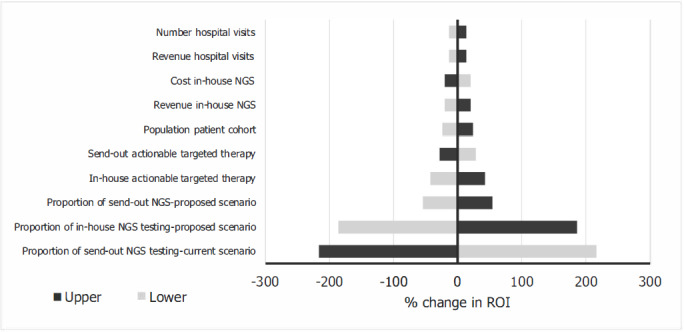
Tornado Diagram of the Results of the One-way Sensitivity Analysis Abbreviations: NGS, next-generation sequencing; ROI, return on investment.

The tornado diagram (**Figure 3**) shows the model input that has the greatest impact on the ROI. The result of the one-way sensitivity analysis showed that the proportions of in-house and send-out NGS testing had the greatest impact on the ROI in the base case.

### Scenario Analysis Results

In the base case, the proposed scenario used in-house NGS testing for 75% of patients. The effect of changing the percentage of in-house NGS testing on the ROI per year are reported in the **Supplementary Material**, as can the changing percentage of in-house NGS testing on the payback period. The ROI was negative, and the payback period was longer than 60 months in the proposed scenario when the percentage of in-house NGS testing was 12% or less. The maximum ROI was $1 423 877 when in-house testing was 100%. The payback period for the scenario with 100% in-house NGS testing was 12 months.

The second scenario analysis varied the number of patients with metastatic NSCLC per year from 50 to 800. The ROI was negative, and the payback period was greater than 60 months when the number of patients was 50. From 100 patients upward, the ROI was positive. At the maximum tested value of 800 patients, the ROI was $1 755 914 and the payback period was 10 months. The yearly ROI and the payback period for the different number of patients can be found in the **Online Supplementary Material**.

For the third scenario, an ROI of $1 972 199 and a payback period of 8 months was estimated for a hospital moving from a 100% single-gene testing in the current scenario to the proposed scenario.

## DISCUSSION

The use of in-house NGS has the potential to increase the revenue and to provide a substantial ROI over a 5-year period in a US hospital with a patient population of 500 and where in-house NGS is used in 75% of these patients.

The use of NGS has been recognized as a cost-effective method for molecular testing. A cost-effectiveness model within the US found that when comparing NGS to single-gene testing, the incremental cost-effectiveness ratio was $7224 per life-year gained.^25^ The use of NGS has also been recognized as a cost-effective method in other settings such as Singapore.^26^

The testing turnaround time is paramount for metastatic NSCLC. The use of fast in-house NGS resulted in an average shorter turnaround time when compared with alternate testing scenarios. A downstream effect of the shorter turnaround time was that more patients could begin with targeted therapy during first-line treatment, reducing the need for an interim therapy. The interim therapies are the nontargeted therapies such as chemotherapy, immunotherapy, or a combination and can be less effective than the targeted therapy.^27^ This occurrence has been investigated in previous studies, which have found that that the long waiting time for test results and consequent delays in treatment initiation can negatively impact patients’ quality of life.^27^ Patients who cannot wait for the biomarker testing are moved to chemotherapy, which may not be the ideal treatment for them. Overall, the effects of long wait times can negatively impact survival.^27^

The faster turnaround time and the prompt start of the appropriate treatment plan as a result of in-house NGS could reduce the number of patients the hospital loses to second opinion. Patients seeking a second opinion are becoming more common in cancer management.^28^ The need for confirmation or certainty and for more personalized information are two factors that can contribute to patients seeking a second opinion.^28^

A higher proportion of patients could be placed on targeted therapy when fast in-house NGS was used. The model also reported that fewer people would be placed on interim therapy when in-house NGS was used. The use of NGS has been shown to increase the use of targeted therapy treatment by 11.9% when compared with single-gene therapy in the US.^25^ In the same study, the number of patients who were biomarker-positive and on an interim therapy decreased by as much as 40.5%. We also identify these trends in the results of our model. An increase in NGS testing in the US in the coming years may be expected as the National Comprehensive Cancer Network (NCCN) decreed that targeted therapy should be used for eligible patients.^29^ Furthermore, it was recommended that patients with advanced NSCLC should have broad molecular testing. The use of targeted therapy as the first-line therapy is beneficial as it is better tolerated by the patient and has a positive response rate.^4,29^

The higher number of patients receiving targeted therapy with the use of fast in-house NGS will also likely have downstream clinical benefits. The use of targeted therapy, according to the NCCN, decreases tumor burden and general symptoms and increases quality of life.^29^ Additionally, a study in Spain reported that actionable mutations were found in 58% of patients and that identifying potential targets ultimately resulted in better outcomes.^30^

Models are by nature a simplified representation of real-life practice. They can provide a likely estimate of reasonable outcomes. The input parameters we use for the base case, though, will likely deviate from those of specific individual hospitals. Hence, as shown by the scenario analyses, the model inputs should be customized so that they accurately reflect the context in which decisions will be made. Model input data collected from different sources constitute a limitation of the study. This is particularly relevant for the costs, where it could be challenging to acquire specific costs. The costs were collected from different studies in which some of the costs were collected from Medicare and others from direct costs. The complexity of the US healthcare system makes it challenging to have costs and a model that can capture this. Other limitations of this model are that the different types of oncogenic drivers or actionable mutations were not explored individually and that the variable turnaround times of different providers based on location and logistical solutions were not considered. The in-house NGS machines could be used in other disease areas, which was not accounted for in the model; further, additional wait times for in-house NGS when the machine was at a maximum capacity due to high volumes were not included. Finally, the therapy-related costs and revenues were not included in the estimations, the inclusion of which would have provided a more comprehensive range of the ROI.

## CONCLUSIONS

The use of a fast in-house NGS testing system instead of sending out the samples could reduce molecular testing turnaround times, increase the proportion of patients on targeted therapy, and potentially reduce a hospitals loss of patients to second opinion. As a further advantage to the hospital, it is expected to increase revenues and provide a positive ROI in a time frame of 12 to 21 months if at least 50% of eligible NSCLC patients received fast in-house NGS testing.

### Author Contributions

U.S., M.B., and R.S. designed and implemented the budget-impact model. A.B.S. and U.S. wrote the manuscript. R.S. and M.B. reviewed the manuscript.

### Disclosures

U.S., M.B., and A.B.S. are employees and R.S. is the owner of Coreva Scientific GmbH & Co KG. Coreva Scientific received consultancy fees for performing, analyzing, and communicating the work presented here.

## Supplementary Material

Online Supplementary Material
