# Supplementary material for: Fast In-House Next-Generation Sequencing in the Diagnosis of Metastatic Non-small Cell Lung Cancer: A Hospital Budget Impact Analysis
Source: J Health Econ Outcomes Res. 2023 Jun 26;10(1):111–8. doi: 10.36469/001c.77686 (PMC10306161; doi:10.36469/001c.77686)
Supplement: Online Supplementary Material [file jheor_2023_10_1_77686_168835.pdf]

Journal of Health Economics and Outcomes Research

Online Supplementary Material

Fast In-House Next-Generation Sequencing in the Diagnosis of Metastatic Non-small Cell Lung Cancer: A Hospital Budget Impact Analysis. *JHEOR*. 2023;10(1):111-118. doi:10.36469/jheor.2023.77686

Table S1: Matrices of the Impact of the Number of Patients and Proportion of In-House NGS on ROI

Table S2: Matrix of the Payback Period for Different Proportions of In-House NGS and Numbers of Patients

Table S3: Number of Patients on Targeted Therapy per Year in the Base Case vs Proposed Scenario

Table S4: CHEERS 2022 Checklist: Reporting Standards for Health Economic Evaluations

This supplementary material has been provided by the authors to give readers additional information about their work.

This is an open-access article distributed under the terms of the Creative Commons Attribution 4.0 International License (CCBY-4.0). View this license's legal deed at http://creativecommons.org/licenses/by/4.0 and legal code at http://creativecommons.org/licenses/by/4.0/legalcode for more information.

Table S1. Matrices of the Impact of the Number of Patients and Proportion of In-House NGS on Return on Investment

Year 1

Proportion of in-house NGS	Number of patients per year: 50	100	200	400	800
5%	- 198,983.98	- 197,967.96	- 195,935.92	- 191,871.83	- 183,743.67
10%	- 197,934.44	- 195,868.89	- 191,737.77	- 183,475.54	- 166,951.09
12%	- 197,514.67	- 195,029.33	- 190,058.67	- 180,117.33	- 160,234.67
15%	- 196,885.05	- 193,770.09	- 187,540.19	- 175,080.38	- 150,160.75
25%	- 194,786.75	- 189,573.50	- 179,147.01	- 158,294.01	- 116,588.03
50%	- 189,545.23	- 179,090.45	- 158,180.90	- 116,361.80	- 32,723.60
100%	- 179,111.66	- 158,223.33	- 116,446.66	- 32,893.32	134,213.37

Year 2

Proportion of in-house NGS	Number of patients per year: 50	100	200	400	800
5%	- 197,683.64	- 195,367.27	- 190,734.55	- 181,469.10	- 162,938.19
10%	- 195,223.85	- 190,447.70	- 180,895.39	- 161,790.78	- 123,581.56
12%	- 194,240.10	- 188,480.19	- 176,960.38	- 153,920.77	- 107,841.53
15%	- 192,764.66	- 185,529.32	- 171,058.63	- 142,117.27	- 84,234.54
25%	- 187,848.40	- 175,696.81	- 151,393.62	- 102,787.23	- 5,574.47
50%	- 175,575.79	- 151,151.59	- 102,303.18	- 4,606.36	190,787.28
100%	- 151,242.37	- 102,484.75	- 4,969.50	190,061.01	580,122.02

Year 3

Proportion of in-house NGS	Number of patients per year: 50	100	200	400	800
5%	- 196,135.24	- 192,270.47	- 184,540.94	- 169,081.89	- 138,163.77
10%	- 191,948.66	- 183,897.33	- 167,794.65	- 135,589.30	- 71,178.60
12%	- 190,274.40	- 180,548.80	- 161,097.61	- 122,195.21	- 44,390.43
15%	- 187,763.44	- 175,526.87	- 151,053.75	- 102,107.49	- 4,214.99
25%	- 179,397.75	- 158,795.50	- 117,591.00	- 35,182.00	129,635.99
50%	- 158,523.98	- 117,047.96	- 34,095.92	131,808.15	463,616.30
100%	- 117,251.66	- 34,503.31	130,993.37	461,986.75	1,123,973.49

Year 4

Proportion of in-house NGS	Number of patients per year: 50	100	200	400	800
5%	- 194,415.29	- 188,830.57	- 177,661.14	- 155,322.28	- 110,644.57
10%	- 188,278.41	- 176,556.81	- 153,113.62	- 106,227.24	- 12,454.48
12%	- 185,824.28	- 171,648.57	- 143,297.14	- 86,594.28	26,811.44
15%	- 182,143.84	- 164,287.67	- 128,575.35	- 57,150.70	85,698.61
25%	- 169,882.88	- 139,765.75	- 79,531.51	40,936.98	281,873.97
50%	- 139,299.88	- 78,599.75	42,800.50	285,601.00	771,202.00
100%	- 78,949.25	42,101.49	284,202.99	768,405.98	1,736,811.96

Year 5

Proportion of in-house NGS	Number of patients per year: 50	100	200	400	800
5%	- 192,574.84	- 185,149.69	- 170,299.37	- 140,598.74	- 81,197.48
10%	- 184,330.29	- 168,660.59	- 137,321.18	- 74,642.36	50,715.28
12%	- 181,033.41	- 162,066.83	- 124,133.65	- 48,267.31	103,465.39
15%	- 176,089.18	- 152,178.36	- 104,356.71	- 8,713.42	182,573.15
25%	- 159,619.08	- 119,238.16	- 38,476.31	123,047.38	446,094.76
50%	- 118,546.81	- 37,093.62	125,812.76	451,625.51	1,103,251.02
100%	- 37,612.27	124,775.46	449,550.92	1,099,101.85	2,398,203.70

Note: The number of patients per year refers to the number of patients added at the beginning of each year.
Abbreviation: NGS, next-generation sequencing.

Table S2. Matrix of the Payback Period for Different Proportions of In-house NGS and Numbers of Patients

Proportion of in-house NGS	Number of patients per year: 50	100	200	400	800
5%	>60	>60	>60	>60	>60
10%	>60	>60	>60	>60	54
12%	>60	>60	>60	>60	45
15%	>60	>60	>60	>60	38
25%	>60	>60	>60	43	25
50%	>60	>60	43	25	14
100%	>60	43	25	14	8

Note: The payback period is the same for the 5-year time horizon in each scenario. The number of patients per year refers to the number of patients added at the beginning of each year.

Table S3. Number of Patients on Targeted Therapy per Year in the Base Case vs Proposed Scenario

	Percentage of Patients on Targeted Therapy		
	**Base Case (%)**	**Proposed (%)**	**Difference**
Year 1	5.7	7.56	1.86
Year 2	8.33	11.05	2.72
Year 3	9.54	12.65	3.12
Year 4	10.09	13.39	3.3
Year 5	10.35	13.73	3.38

Table S4. CHEERS 2022 Checklist[1]: Reporting Standards for Health Economic Evaluations

Selection/Item	Recommendation	Page/Line[a]
**Title and abstract**		
1. Title	Identify the study as an economic evaluation and specify the interventions being compared.	1/1-3
2. Abstract	Provide a structured summary that highlights context, key methods, results, and alternative analyses.	1/4-2/33
**Introduction**		
3. Background and objectives	Give the context for the study, the study question, and its practical relevance for decision making in policy or practice.	3/36-4/67
**Methods**		
4. Health economic analysis plan	Indicate whether a health economic analysis plan was developed and where available.	–
5. Study population	Describe characteristics of the study population (such as age range, demographics, socioeconomic, or clinical characteristics).	4/72-73
6. Setting and location	Provide relevant contextual information that may influence findings.	4/70-75
7. Comparators	Describe the interventions or strategies being compared and why chosen.	4/73-74
8. Perspective	State the perspective(s) adopted by the study and why chosen.	4/70-71
9. Time horizon	State the time horizon for the study and why appropriate.	4/74-75
10. Discount rate	Report the discount rate(s) and reason chosen.	20/398-399
11. Selection of outcomes	Describe what outcomes were used as the measure(s) of benefit(s) and harm(s).	7/136-141
12. Measurement of outcomes	Describe how outcomes used to capture benefit(s) and harm(s) were measured.	7/137- 8/160
13. Valuation of outcomes	Describe the population and methods used to measure and value outcomes.	4/70-73;7/130-135
14. Measurement and valuation of resources and costs	Describe how costs were valued.	6/135
15. Currency, price date, and conversion	Report the dates of the estimated resource quantities and unit costs, plus the currency and year of conversion.	6/135
16. Rationale and description of model	If modeling is used, describe in detail, and why used. Report if the model is publicly available and where it can be accessed.	4/78-8/160
17. Analytics and assumptions	Describe any methods for analyzing or statistically transforming data, any extrapolation methods, and approaches for validating any model used.	7/130-8/160
18. Characterizing heterogeneity	Describe any methods used for estimating how the results of the study vary for subgroups.	7/149-8/160
19. Characterizing distributional effects	Describe how impacts are distributed across different individuals or adjustments made to reflect priority populations.	–
20. Characterizing uncertainty	Describe methods to characterize any sources of uncertainty in the analysis.	7/149-8/160
21. Approach to engagement with patients and others affected by the study	Describe any approaches to engage patients or service recipients, the general public, communities, or stakeholders (such as clinicians or payers) in the design of the study.	–

Table S4. CHEERS 2022 Checklist[a]: Reporting Standards for Health Economic Evaluations, *cont'd*

**Results**		
22. Study parameters	Report all analytic inputs (such as values, ranges, references) including uncertainty or distributional assumptions.	8/163-9/197
23. Summary of main results	Report the mean values for the main categories of costs and outcomes of interest and summarize them in the most appropriate overall measure.	8/163-9/197
24. Effect of uncertainty	Describe how uncertainty about analytic judgments, inputs, or projections affect findings. Report the effect of choice of discount rate and time horizon, if applicable.	9/189-10/205
25. Effect of engagement with patients and others affected by the study	Report on any difference patient/service recipient, general public, community, or stakeholder involvement made to the approach or findings of the study.	–
**Discussion**		
26. Study findings, limitations, generalizability, and current knowledge	Report key findings, limitations, ethical or equity considerations not captured, and how these could affect patients, policy, or practice.	10/207-12/262
**Other relevant information**		
27. Source of funding	Describe how the study was funded and any role of the funder in the identification, design, conduct, and reporting of the analysis.	13/275
28. Conflicts of interest	Report authors conflicts of interest according to journal or International Committee of Medical Journal Editors requirements.	13/271-273

[a]References to page and line numbers in the original manuscript.

REFERENCE

1. Consolidated Health Economic Evaluation Reporting Standards (CHEERS) 2022 explanation and elaboration: a report of the ISPOR CHEERS II Good Practices Task Force. *Value Health*. 2022;25(6):1060. doi:10.1016/j.jval.2022.03.002.
